# An Analysis of Factors Influencing Comorbid Anxiety and Depression Among Adults in China: A Large‐Scale Cross‐Sectional Study

**DOI:** 10.1002/brb3.71843

**Published:** 2026-10-21

**Authors:** Zhuoqun Chen, Jiaying Huang, Yuying Wang, Jingxian Chen, Ke Zhu, Tongjie Chen, Xin‐lin Chen, Wenyan Tan

**Affiliations:** ^1^ School of Basic Medical Sciences Guangzhou University of Chinese Medicine Guangzhou China; ^2^ Guangdong Mental Health Center Guangdong Provincial People's Hospital (Guangdong Academy of Medical Sciences) Southern Medical University Guangzhou China

**Keywords:** comorbid anxiety and depression, cross‐sectional study, decision tree, influencing factors

## Abstract

**Background:**

Comorbid anxiety and depression (CAD) are common, but the factors influencing this comorbidity have not been comprehensively studied. The aim of this study was to investigate the associations of sociodemographic and behavioral factors with CAD among residents of Guangdong Province in China.

**Methods:**

A cross‐sectional study was conducted, and questionnaires, including the Depression Screening Scale (PHQ‐9), the Anxiety Screening Scale (GAD‐7), and the Insomnia Severity Index (ISI), were distributed among permanent residents aged 18 years and above in Guangdong Province. Logistic regression and decision tree modeling were used to construct a model to identify factors associated with CAD in adults.

**Results:**

Based on a sample of 51,774 participants from the Guangdong Provincial Mental Health Database, four groups were compared: healthy (*n* = 48,657), anxiety alone (*n* = 285), depression alone (*n* = 1677), and CAD (*n* = 1155). Factors such as sex, age, district, insomnia, BMI, education, marital status, income, smoking, drinking, dietary regularity, exercise frequency, electronic device use, daily steps, sedentary time, and physical chronic diseases were observed significant differences across four groups (*p* < 0.05). In univariable analyses, factors associated with CAD (vs. healthy) included female sex, younger age, urban residence, insomnia, irregular diet, prolonged electronic device use, prolonged sedentary time, and lack of exercise. Multivariable analysis revealed that insomnia (OR = 14.64, 95% CI: 12.49–17.15), irregular diet (OR = 2.96, 95% CI: 2.45–3.58), prolonged electronic device use (OR = 1.78, 95% CI: 1.51–2.11), physical chronic diseases (OR = 1.59, 95% CI: 1.38–1.82), and female (OR = 1.19, 95% CI: 1.04–1.37) were independently associated with higher risk of CAD (all *p* < 0.05). Older age, married/cohabiting status, higher income, occasionally regular exercise, moderate steps, and moderate sedentary time were protective factors (ORs 0.41–0.80, all *p* < 0.05). District, smoking, and BMI were not retained in the multivariable model. The logistic regression model showed an AUC of 0.866 (95% CI: 0.845–0.886), and the decision tree model showed an AUC of 0.839 (95% CI: 0.818–0.860). The Hosmer–Lemeshow test indicated good calibration for the logistic model (*χ* ^2^ = 2.09, df = 3, *p* = 0.553).

**Conclusion:**

This large cross‐sectional study indicated that insomnia, irregular diet, prolonged device use, and physical chronic diseases were associated with an increased risk of CAD. The logistic regression model showed good discriminative ability, and the decision tree served as an exploratory tool for visualizing risk stratification. Further prospective studies are needed to assess predictive utility.

## Graphical abstract

In 51,774 Chinese adults, insomnia, irregular diet, prolonged device use, and physical chronic disease were associated with higher odds of comorbid anxiety and depression, whereas older age, marriage, and moderate daily steps were protective. Screening for insomnia and modifiable lifestyle factors may help identify those at risk.

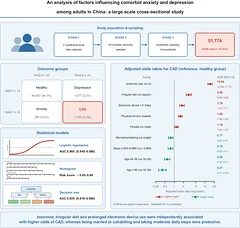

## 1 Introduction

In the 21st century, the global health toll from psychiatric diagnosis remains enormous; there in comorbidity is a common and pervasive phenomenon (GBD 2019 Mental Disorders Collaborators 2022; Ter Meulen et al. 2021). Comorbidity is defined as a condition that exists or occurs in the course of a disease other than the one in which the same patient suffers (Y. Wu et al. 2025). Anxiety and depression often coexist, and comorbidity between anxiety and depression is one of the most common psychiatric comorbidities, known as “comorbid anxiety and depression” (CAD) (Huang et al. 2019). Another review estimates that about 85% of individuals with depressive symptoms reported comorbid anxiety, and up to 90% of those with anxiety reported depressive symptoms (Choi et al. 2020). Anxiety affects 50%–75% of people with major depressive disorder (Su 2023), whereas 30%–63% of patients with anxiety disorders exhibit symptoms of major depressive disorder (Su 2023; Harrison et al. 2021; Davies et al. 2023). In China, 70%–80% of patients with depressive disorders have different degrees of anxiety, indicating a higher prevalence of comorbidity (W. Zhang et al. 2025).

CAD patients are associated with more severe health outcomes, a greater risk of chronic disease, and greater dysfunction than patients with only one of these conditions (Groen et al. 2020; Liu et al. 2022), as well as poorer general adjustment and family relationships, lower life satisfaction, increased chronic stress (Essau et al. 2018), and even an increased prevalence of frailty among elderly Chinese individuals (Zhao et al. 2020). Given that the treatment for CAD is more complex than a single disorder (Furukawa et al. 2018), it has a profound impact on family and social relationships, resulting in a heavier medical‐economic burden, greater risks to social security, and added strain on the public health system. Therefore, it is important to identify key modifiable factors associated with CAD to inform targeted prevention and intervention strategies that may help reduce the prevalence of the condition, facilitate its early recognition, and avoid underdiagnosis or misdiagnosis.

For example, a large‐scale meta‐analysis of 201 studies involving nearly 198,000 medical students during the COVID‐19 pandemic reported a high prevalence of depression (41%) and anxiety (38%), and identified risk factors such as female sex, young age, academic stress, and low physical activity (Peng et al. 2023). A study in western China that included 6838 individuals aged 50 years and older reported a CAD incidence of 8.3%. The combination group was characterized by a greater proportion of women, lower education levels, a higher prevalence of single/divorced/widowed individuals, higher rates of alcohol consumption and smoking, a greater burden of chronic disease, and cognitive decline (Liu et al. 2022). In several cross‐sectional studies of a national population of college students, length of time on electronic devices, sleep disturbances, and physical activity were found to be risk factors for CAD (Qu et al. 2021; Niu et al. 2023; M. Wang, Tao, et al. 2023). Although numerous studies have investigated the risk factors of comorbidity, most have concentrated on specific populations or small samples , leaving relatively few studies focused on the general population.

In China, CAD is very common among adolescents (M. Wang, Mou, et al. 2023), but few studies have examined its prevalence and associated factors in the general adult population. Using data from a large‐scale epidemiological survey in Guangdong Province, this study aimed to identify factors associated with CAD—including socioeconomic status, chronic disease burden, sleep quality, and health behaviors—using logistic regression and a decision tree as an exploratory visualization tool. These findings may serve as a foundation for future longitudinal or mechanistic investigations into the potential causal pathways of CAD.

## 2 Materials and Methods

### 2.1 Study Design and Sample Selection

This study is a cross‐sectional investigation executed under the guidance and supervision of the Guangdong Mental Health Center. A multistage stratified equal‐probability sampling was carried out between October 2022 and December 2022, covering residents aged 18 years or above (born before September 1, 2005, and having resided in Guangdong Province for more than 6 months) from 21 prefecture‐level cities in Guangdong Province.

According to the multistage stratified equal probability random sampling method, three districts (cities and counties) were randomly selected from each prefecture, and three streets/towns were randomly selected from each district (cities and counties). A total of 189 streets/towns and counties were selected. Two village (residential) committees were randomly selected from each street/town, and 111–112 households were selected from each neighborhood committee according to a systematic sampling method (such as the starting point and spacing determined by the door number) for investigation. The minimum sample size of each prefecture‐level city is 2000 cases, and a total of 51,774 samples were collected in the province. Stratified sampling was conducted according to age and sex, and the male‐to‐female ratio was required to be no less than 0.9:1.

### 2.2 Inclusion and Exclusion Criteria

The inclusion criteria were samples over 18 years of age (including 18 years of age), local permanent residents (permanent residents who have resided for ≥ 6 months and are living permanently), and those who voluntarily participated in the program and who signed an informed consent form. The exclusion criteria were participants who lacked the ability to self‐assess or refused to participate, and non‐Guangdong household participants.

### 2.3 Measurements

Questionnaire design: The questionnaire used in this study was developed by the Guangdong Mental Health Center. The questionnaire included screening for sociodemographic information (gender, age, education, marital status, education level, occupation, etc.), behavioral factors (smoking, alcohol consumption, diet, physical activity, etc.), and chronic diseases.

The Patient Health Questionnaire‐9 (PHQ‐9) was selected for depression screening, and the PHQ‐9 is specifically designed to assess symptoms of depression. It consists of nine items that meet the DSM‐IV diagnostic criteria for depression and is a concise questionnaire. Each item of PHQ‐9 is rated on a 4‐point scale (0 = not at all, 1 = several days, 2 = more than half the days, 3 = nearly every day), yielding a total score ranging from 0 to 27.Patient depression outcomes were judged according to the scoring options, and formal validation of the PHQ‐9 included a comparison with a structured diagnostic interview conducted by a mental health professional, with specificity and sensitivity of 88% and 88%, respectively, in identifying major depression when a score of ≥ 10 on the PHQ‐9 was selected as positive for depression. The Chinese version of the PHQ‐9 for this investigation has been experimentally and comprehensively validated, demonstrating that it has strong psychometric properties and can be used to screen populations for depression (Kroenke et al. 2001). In this study, residents who scored 10 points or more on the PHQ‐9 scale were considered to meet the diagnostic criteria for positive depression.

The Generalized Anxiety Disorder‐7 (GAD‐7) was selected for screening for anxiety disorders. The GAD‐7 is specifically designed to assess generalized anxiety in primary care settings and consists of seven items that allow for a rapid assessment of the frequency and severity of a respondent's anxiety symptoms over the past 2 weeks. The GAD‐7 comprises individual items, each of which has a score ranging from 0 (mild) to 3 (severe), with a total score ranging from 0 to 21. In a formal validation against the Diagnostic Psychiatric Interview, selecting a GAD‐7 score ≥10 as anxiety‐positive had a specificity of 82% and a sensitivity of 89% in detecting generalized anxiety disorders. The GAD‐7 has demonstrated excellent retest reliability and validity in surveys of individuals of different ages (Spitzer et al. 2006). In this research, residents with a GAD‐7 score of 10 points or more were regarded as meeting the diagnostic criteria for positive anxiety.

Insomnia symptoms were assessed using the Insomnia Severity Index (ISI) (Morin et al. 2011), a widely used 7‐item self‐report questionnaire designed to evaluate the nature, severity, and impact of insomnia over the past 2 weeks. Each item is rated on a 5‐point Likert scale from 0 (no problem) to 4 (very severe problem), yielding a total score ranging from 0 to 28, with higher scores indicating more severe insomnia symptoms. The Chinese version of the ISI has been validated in a community‐dwelling older Chinese population, demonstrating satisfactory internal consistency (Cronbach's *α* = 0.81) (D. S. Yu 2010). In this study, a total ISI score of ≥ 8 was used to define the presence of insomnia, as recommended by previous validation studies. The binary variable (insomnia vs. no insomnia) was entered into both the logistic regression models and the decision tree analysis.

Physical chronic disease was assessed using a single multiple‐choice questionnaire item, G15: “In the past year, have you been clinically diagnosed with any of the following chronic diseases? (Multiple choices allowed).” The full wording of item G15 and the coding rules are provided in Box S1. Because the outcome of this study included anxiety and depression, mental and sleep‐related conditions were excluded from the primary exposure definition to avoid circularity and overlap with the outcome. Specifically, the following G15 items were excluded: insomnia (5), obstructive sleep apnea syndrome (6), narcolepsy/hypersomnia (9), depression (19), anxiety disorder (20), bipolar disorder (21), schizophrenia (22), sleep disorder (23), post‐traumatic stress disorder (24), and panic disorder (25). Participants who selected at least one of the remaining physical chronic conditions were coded as 1. Participants who did not select any remaining physical chronic condition were coded as 0.

### 2.4 Definition of CAD

Participants with a PHQ‐9 score ≥ 10 and a GAD‐7 score ≥ 10 were classified as having CAD. Those with PHQ‐9 < 10 and GAD‐7 < 10 were considered healthy. Participants who met only one of the two criteria were classified as depression alone or anxiety alone.

### 2.5 Statistical Analysis

Statistical analyses were performed using R software (version 4.4.1). Normality was assessed with the Kolmogorov–Smirnov test. Normally distributed continuous data were presented as means ± SD and compared using one‐way ANOVA; non‐normally distributed data were presented as median (P25, P75) and compared using the Kruskal–Wallis test. Categorical variables were expressed as frequencies (%) and compared using the chi‐square test.

Univariable logistic regression was first performed to screen factors associated with CAD. Variables with a univariable *p* < 0.10 were entered into a multivariable logistic regression model. The final model was built using a forward stepwise selection procedure (entry *p* < 0.05, removal *p* > 0.10). For categorical predictors with more than two levels, the overall effect was assessed with the Type III Wald chi‐square test (results shown in Table S1). Before constructing the multivariable model, we assessed multicollinearity among the independent variables by calculating the variance inflation factor (VIF) and examining pairwise correlation matrices. The final multivariable model was used to construct a nomogram and to generate ROC curves and calibration curves; the Hosmer–Lemeshow test was used to evaluate calibration.

A separate decision tree model was developed using the classification and regression tree (CART) algorithm, including all independent variables. The minimum number of cases for parent and child nodes was set to 400 and 100, respectively. Splitting and merging were tested at *α* = 0.05. Variable importance was measured as the total decrease in node impurity (Gini index) attributable to each predictor across all splits in the tree. Importance values were then scaled to a relative importance with the largest value set to 100%. For both models, a two‐sided *p* < 0.05 was considered statistically significant unless otherwise specified.

To examine whether residual overlap between the exposure and the outcome influenced the findings, a sensitivity analysis was performed in which all participants reporting at least one of the ten mental or sleep‐related conditions were excluded, irrespective of whether they also reported a physical chronic condition (*n* = 3510; 1469 with both physical and mental or sleep‐related conditions and 2041 with mental or sleep‐related conditions only). The multinomial logistic regression model was then refitted in the remaining 48,264 participants with an identical set of covariates and the same reference category (healthy participants), and the estimates were compared with those of the primary analysis.

## 3 Results

### 3.1 Baseline Characteristics

The study included 51,774 participants (Figure 1), consisting of 285 with anxiety alone, 1677 with depression alone, 1155 with CAD, and 48,657 healthy controls (Table 1).

**FIGURE 1:**
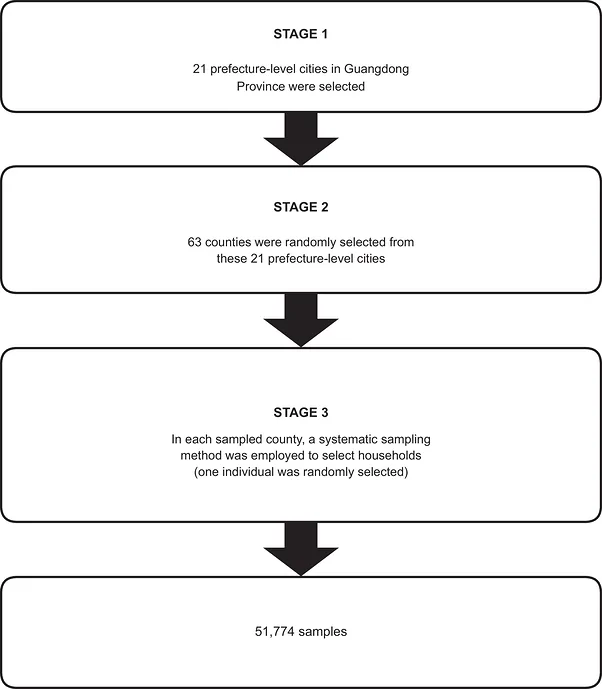
Flow chart of the sampling procedure in this study.

**TABLE 1:** Sociodemographic characteristics and health‐related factors of participants.

Variables	Total (*n* = 51,774)	Healthy (*n* = 48,657)	Anxiety (*n* = 285)	Depression (*n* = 1677)	Comorbidity (*n* = 1155)	Statistic	*p*
**Gender, *n* (%)**						*χ* ^2^ = 56.69	< 0.001
Male	24,181 (46.70)	22,921 (47.11)	99 (34.74)	690 (41.14)	471 (40.78)		
Female	27,593 (53.30)	25,736 (52.89)	186 (65.26)	987 (58.86)	684 (59.22)		
**Age, *n* (%)**						*χ* ^2^ = 668.21	< 0.001
18–29	15,614 (30.16)	14,105 (28.99)	102 (35.79)	823 (49.08)	584 (50.56)		
30–44	13,175 (25.45)	12,364 (25.41)	95 (33.33)	433 (25.82)	283 (24.50)		
45–59	14,549 (28.10)	13,989 (28.75)	63 (22.11)	297 (17.71)	200 (17.32)		
≥ 60	8436 (16.29)	8199 (16.85)	25 (8.77)	124 (7.39)	88 (7.62)		
**District, *n* (%)**						*χ* ^2^ = 140.85	< 0.001
Urban	31,802 (61.42)	29,577 (60.79)	199 (69.82)	1183 (70.54)	843 (72.99)		
Rural	19,972 (38.58)	19,080 (39.21)	86 (30.18)	494 (29.46)	312 (27.01)		
**Insomnia, *n* (%)**						*χ* ^2^ = 5662.37	< 0.001
No	39,638 (76.56)	38,971 (80.09)	98 (34.39)	371 (22.12)	198 (17.14)		
Yes	12,136 (23.44)	9686 (19.91)	187 (65.61)	1306 (77.88)	957 (82.86)		
**BMI, *n* (%)**						*χ* ^2^ = 117.04	< 0.001
< 18.5 kg/m^2^	3799 (7.34)	3440 (7.07)	30 (10.53)	179 (10.67)	150 (12.99)		
18.5–24 kg/m^2^	30,321 (58.56)	28,610 (58.80)	159 (55.79)	944 (56.29)	608 (52.64)		
24–28 kg/m^2^	14,372 (27.76)	13,571 (27.89)	76 (26.67)	435 (25.94)	290 (25.11)		
≥ 28 kg/m^2^	3282 (6.34)	3036 (6.24)	20 (7.02)	119 (7.10)	107 (9.26)		
**Educational level**						*χ* ^2^ = 474.39	< 0.001
Elementary school or below	21,134 (40.82)	20,380 (41.89)	86 (30.18)	388 (23.14)	280 (24.24)		
Secondary education	12,230 (23.62)	11,479 (23.59)	58 (20.35)	420 (25.04)	273 (23.64)		
University or above	18,410 (35.56)	16,798 (34.52)	141 (49.47)	869 (51.82)	602 (52.12)		
**Marital status**						*χ* ^2^ = 625.28	< 0.001
Single	7782 (15.03)	6859 (14.10)	53 (18.60)	519 (30.95)	351 (30.39)		
Married/cohabiting	41,781 (80.70)	39,764 (81.72)	221 (77.54)	1066 (63.57)	730 (63.20)		
Divorced/widowed	2211 (4.27)	2034 (4.18)	11 (3.86)	92 (5.49)	74 (6.41)		
**Monthly per capita household income (RMB)**						*χ* ^2^ = 24.34	< 0.001
< 3500	20,509 (39.61)	19,344 (39.76)	98 (34.39)	646 (38.52)	421 (36.45)		
3500–6000	18,181 (35.12)	17,115 (35.17)	94 (32.98)	553 (32.98)	419 (36.28)		
≥ 6000	13,084 (25.27)	12,198 (25.07)	93 (32.63)	478 (28.50)	315 (27.27)		
**Smoking habit**						*χ* ^2^ = 8.38	0.039
No	39,150 (75.62)	36,743 (75.51)	233 (81.75)	1293 (77.10)	881 (76.28)		
Yes	12,624 (24.38)	11,914 (24.49)	52 (18.25)	384 (22.90)	274 (23.72)		
**Drinking habit**						*χ* ^2^ = 33.06	< 0.001
No	42,088 (81.29)	39,667 (81.52)	233 (81.75)	1306 (77.88)	882 (76.36)		
Yes	9686 (18.71)	8990 (18.48)	52 (18.25)	371 (22.12)	273 (23.64)		
**Dietary regularity**						*χ* ^2^ = 1631.63	< 0.001
Regularly	46,417 (89.65)	44,195 (90.83)	226 (79.30)	1213 (72.33)	783 (67.79)		
More regular	3595 (6.94)	3144 (6.46)	34 (11.93)	252 (15.03)	165 (14.29)		
Irregularly	1762 (3.40)	1318 (2.71)	25 (8.77)	212 (12.64)	207 (17.92)		
**Exercise frequency**						*χ* ^2^ = 300.53	< 0.001
Never	27,816 (53.73)	25,685 (52.79)	175 (61.40)	1146 (68.34)	810 (70.13)		
Occasionally	14,887 (28.75)	14,233 (29.25)	65 (22.81)	351 (20.93)	238 (20.61)		
Often	9071 (17.52)	8739 (17.96)	45 (15.79)	180 (10.73)	107 (9.26)		
**Average daily electronic device usage time**						*χ* ^2^ = 1008.25	< 0.001
< 4 h	41,211 (79.60)	39,345 (80.86)	199 (69.82)	1012 (60.35)	655 (56.71)		
4–6 h	5773 (11.15)	5245 (10.78)	47 (16.49)	289 (17.23)	192 (16.62)		
> 6 h	4790 (9.25)	4067 (8.36)	39 (13.68)	376 (22.42)	308 (26.67)		
**Average daily walking steps per day**						*χ* ^2^ = 55.42	< 0.001
≤ 2999 steps	33,291 (64.30)	31,110 (63.94)	188 (65.96)	1168 (69.65)	825 (71.43)		
3000–6999 steps	8885 (17.16)	8408 (17.28)	44 (15.44)	273 (16.28)	160 (13.85)		
≥ 7000 steps	9598 (18.54)	9139 (18.78)	53 (18.60)	236 (14.07)	170 (14.72)		
**Average daily sedentary time**						*χ* ^2^ = 639.53	< 0.001
< 1 h	24,427 (47.18)	23,407 (48.11)	105 (36.84)	530 (31.60)	385 (33.33)		
1–5 h	15,549 (30.03)	14,717 (30.25)	80 (28.07)	486 (28.98)	266 (23.03)		
≥ 6 h	11,798 (22.79)	10,533 (21.65)	100 (35.09)	661 (39.42)	504 (43.64)		
**Physical chronic diseases** ^a^						*χ* ^2^ = 172.61	< 0.001
No	38,750 (74.84)	36,721 (75.47)	181 (63.51)	1119 (66.73)	729 (63.12)		
Yes	13,024 (25.16)	11,936 (24.53)	104 (36.49)	558 (33.27)	426 (36.88)		

Abbreviation: *χ* ^2^, chi‐square test.

^a^Physical chronic diseases, defined as any self‐reported, physician‐diagnosed condition in item G15 after excluding 10 mental and sleep‐related conditions (insomnia, obstructive sleep apnea syndrome, narcolepsy/hypersomnia, depression, anxiety disorder, bipolar disorder, schizophrenia, sleep disorder, post‐traumatic stress disorder, and panic disorder).

Compared with the healthy group, the CAD group had a higher proportion of females (59.22% vs. 52.89%), younger individuals (75.06% aged under 45 years vs. 54.40%), urban residents (72.99% vs. 60.79%), and participants with insomnia (82.86% vs. 19.91%). The CAD group also showed higher rates of university education or above (52.12% vs. 34.52%), irregular diet (17.92% vs. 2.71%), low exercise frequency (70.13% never exercised vs. 52.79%), and prolonged electronic device use (> 6 h/day: 26.67% vs. 8.36%). Notably, a higher percentage of the CAD group reported physical chronic diseases (36.88% vs. 24.53%), including cardiovascular disease, cerebrovascular disease, arthritis, and so on (Box S1 and Table S6).

### 3.2 Logistic Regression

Tables 2 and 3 present the results of the univariable and multivariable logistic regression analyses, respectively. In the final multivariable model, no multicollinearity was detected (all GVIF values were below 2.2; Table S2).

**TABLE 2:** Univariable logistic regression analysis of characteristics associated with depression, anxiety, and CAD.

Variables		Comorbidity vs. healthy		Anxiety vs. healthy		Depression vs. healthy	
		OR(95% CI)	*p* value	OR(95% CI)	*p* value	OR(95% CI)	*p* value
**Gender**	Male	Ref		Ref		Ref	
	Female	1.29 (1.15–1.46)	< 0.001	1.67 (1.31–2.14)	< 0.001	1.27 (1.15–1.41)	< 0.001
**Age**	18–29	Ref		Ref		Ref	
	30–44	0.55 (0.48–0.64)	< 0.001	1.06 (0.80–1.41)	0.672	0.60 (0.53–0.68)	< 0.001
	45–59	0.35 (0.29–0.41)	< 0.001	0.62 (0.45–0.85)	0.003	0.36 (0.32–0.42)	< 0.001
	≥ 60	0.26 (0.21–0.32)	< 0.001	0.42 (0.27–0.65)	< 0.001	0.26 (0.21–0.31)	< 0.001
**District**	Urban	Ref		Ref		Ref	
	Rural	0.57 (0.50–0.65)	< 0.001	0.67 (0.52–0.86)	0.002	0.65 (0.58–0.72)	< 0.001
**Insomnia**	No	Ref		Ref		Ref	
	Yes	19.45 (16.66–22.70)	< 0.001	7.68 (6.01–9.81)	< 0.001	14.16 (12.59–15.93)	< 0.001
**BMI**	< 18.5 kg/m^2^	Ref		Ref		Ref	
	18.5–24 kg/m^2^	0.49 (0.41–0.58)	< 0.001	0.64(0.43–0.95)	0.025	0.63 (0.54–0.75)	< 0.001
	24–28 kg/m^2^	0.49 (0.40–0.60)	< 0.001	0.64 (0.42–0.98)	0.042	0.62 (0.52–0.74)	< 0.001
	≥ 28 kg/m^2^	0.81 (0.63–1.05)	0.101	0.76 (0.43–1.34)	0.343	0.75 (0.59–0.96)	0.019
**Educational level**	Elementary school or below	Ref		Ref		Ref	
	Secondary education	1.73 (1.46–2.05)	< 0.001	1.20 (0.86–1.67)	0.290	1.92 (1.67–2.21)	< 0.001
	University or above	2.61 (2.26–3.01)	< 0.001	1.99 (1.52–2.60)	< 0.001	2.72 (2.41–3.07)	< 0.001
**Marital status**	Single	Ref		Ref		Ref	
	Married/cohabiting	0.36 (0.32–0.41)	< 0.001	0.72 (0.53–0.97)	0.032	0.35 (0.32–0.39)	< 0.001
	Divorced/widowed	0.71 (0.55–0.92)	0.009	0.70 (0.36–1.34)	0.283	0.60 (0.48‐0.75)	< 0.001
**Monthly per capita household income (RMB)**	< 3500	Ref		Ref		Ref	
	3500–6000	1.13 (0.98–1.29)	0.092	1.08 (0.82–1.44)	0.584	0.97 (0.86–1.09)	0.576
	≥ 6000	1.19 (1.02–1.38)	0.023	1.50 (1.13–2.00)	0.005	1.17 (1.04–1.32)	0.009
**Smoking habit**	No	Ref		Ref		Ref	
	Yes	0.96 (0.84–1.10)	0.551	0.69 (0.51–0.93)	0.015	0.92 (0.82–1.03)	0.137
**Drinking habit**	No	Ref		Ref		Ref	
	Yes	1.37 (1.19–1.57)	< 0.001	0.98 (0.73–1.33)	0.92	1.25 (1.11–1.41)	< 0.001
**Dietary regularity**	Regularly	Ref		Ref		Ref	
	More regular	2.96 (2.49–3.52)	< 0.001	2.11 (1.47–3.04)	< 0.001	2.92 (2.54–3.36)	< 0.001
	Irregularly	8.86 (7.53–10.43)	< 0.001	3.71 (2.45–5.63)	< 0.001	5.86 (5.01–6.85)	< 0.001
**Exercise frequency**	Never	Ref		Ref		Ref	
	Occasionally	0.53 (0.46–0.61)	< 0.001	0.67 (0.50–0.89)	0.006	0.55 (0.49–0.62)	< 0.001
	Often	0.39 (0.32–0.48)	< 0.001	0.76 (0.54–1.05)	0.095	0.46 (0.39–0.54)	< 0.001
**Average daily electronic device usage time**	< 4 h	Ref		Ref		Ref	
	4–6 h	2.20 (1.87–2.59)	< 0.001	1.77 (1.29–2.44)	< 0.001	2.14 (1.87–2.45)	< 0.001
	> 6 h	4.55 (3.96–5.23)	< 0.001	1.90 (1.34–2.68)	< 0.001	3.59 (3.18–4.06)	< 0.001
**Average daily walking steps per day**	≤ 2999 steps	Ref		Ref		Ref	
	3000–6999 steps	0.72 (0.61–0.85)	< 0.001	0.87 (0.62–1.20)	0.394	0.86 (0.76–0.99)	0.034
	≥ 7000 steps	0.70 (0.59–0.83)	< 0.001	0.96 (0.71–1.31)	0.804	0.69 (0.60–0.79)	< 0.001
**Average daily sedentary time**	< 1 h	Ref		Ref		Ref	
	1–5 h	1.10 (0.94–1.29)	0.243	1.21 (0.91–1.62)	0.196	1.46 (1.29–1.65)	< 0.001
	≥ 6 h	2.91 (2.54–3.33)	< 0.001	2.12 (1.61–2.79)	< 0.001	2.77 (2.47–3.11)	< 0.001
**Chronic diseases**	No	Ref		Ref		Ref	
	Yes	1.80 (1.59–2.03)	< 0.001	1.77 (1.39–2.25)	< 0.001	1.53 (1.38–1.70)	< 0.001

**TABLE 3:** Multivariable logistic regression analysis of characteristics associated with depression, anxiety, and CAD.

Variables		Comorbidity vs. healthy		Anxiety vs. healthy		Depression vs. healthy	
		OR(95% CI)	*p* value	OR(95% CI)	*p* value	OR(95% CI)	*p* value
**Gender**	Male	Ref		Ref		Ref	
	Female	1.19 (1.04–1.37)	0.014	1.58 (1.20–2.08)	0.001	1.17 (1.05–1.32)	0.007
**Age**	18–29	Ref		Ref		Ref	
	30–44	0.75 (0.63–0.90)	0.001	1.17 (0.85–1.62)	0.327	0.84 (0.73–0.97)	0.017
	45–59	0.59 (0.48–0.73)	< 0.001	0.76 (0.52–1.12)	0.173	0.62 (0.53–0.74)	< 0.001
	≥ 60	0.41 (0.31–0.55)	< 0.001	0.48 (0.28–0.82)	0.007	0.42 (0.33–0.53)	< 0.001
**Insomnia**	No	Ref		Ref		Ref	
	Yes	14.64 (12.49–17.15)	< 0.001	6.42 (4.98–8.27)	< 0.001	11.22 (9.94–12.67)	< 0.001
**Educational level**	Elementary school or below	Ref		Ref		Ref	
	Secondary education	1.19 (0.99–1.44)	0.064	0.93 (0.65–1.32)	0.682	1.38 (1.18–1.61)	< 0.001
	University or above	1.21 (1.01–1.45)	0.041	1.13 (0.81–1.58)	0.477	1.33 (1.14–1.55)	< 0.001
**Marital status**	Single	Ref		Ref		Ref	
	Married/cohabiting	0.77 (0.65–0.91)	0.002	0.99 (0.69–1.43)	0.976	0.68 (0.59–0.78)	< 0.001
	Divorced/widowed	1.25 (0.93–1.68)	0.145	0.82 (0.41–1.65)	0.579	0.99 (0.76–1.29)	0.963
**Monthly per capita household income (RMB)**	< 3500	Ref		Ref		Ref	
	3500–6000	0.87 (0.75–1.01)	0.062	0.93 (0.69–1.25)	0.615	0.73 (0.64–0.82)	< 0.001
	≥ 6000	0.76 (0.64–0.90)	0.001	1.09 (0.79–1.49)	0.604	0.73 (0.64–0.84)	< 0.001
**Drinking habit**	No	Ref		Ref		Ref	
	Yes	1.23 (1.04–1.45)	0.013	1.09 (0.77–1.53)	0.635	1.18 (1.02–1.35)	0.022
**Dietary regularity**	Regularly	Ref		Ref		Ref	
	More regular	1.55 (1.28–1.87)	< 0.001	1.56 (1.07–2.28)	0.021	1.58 (1.36–1.85)	< 0.001
	Irregularly	2.96 (2.45–3.58)	< 0.001	2.08 (1.34–3.24)	0.001	2.11 (1.77–2.52)	< 0.001
**Exercise frequency**	Never	Ref		Ref		Ref	
	Occasionally	0.71 (0.61–0.83)	< 0.001	0.74 (0.55–1.00)	0.049	0.68 (0.60–0.77)	< 0.001
	Often	0.72 (0.58–0.90)	0.004	1.07 (0.74–1.53)	0.726	0.82 (0.69–0.98)	0.026
**Average daily electronic device usage time**	< 4 h	Ref		Ref		Ref	
	4–6 h	1.25 (1.04–1.49)	0.016	1.19 (0.85–1.67)	0.310	1.21 (1.05–1.41)	0.010
	> 6 h	1.78 (1.51–2.11)	< 0.001	0.98 (0.67–1.43)	0.904	1.53 (1.32–1.77)	< 0.001
**Average daily walking steps per day**	≤ 2999 steps	Ref		Ref		Ref	
	3000–6999 steps	0.76 (0.63–0.91)	0.003	0.85 (0.60–1.19)	0.330	0.88 (0.76–1.02)	0.081
	≥ 7000 steps	0.86 (0.72–1.03)	0.104	1.05 (0.76–1.46)	0.764	0.80 (0.69–0.94)	0.005
**Average daily sedentary time**	< 1 h	Ref		Ref		Ref	
	1–5 h	0.80 (0.68–0.95)	0.009	0.98 (0.72–1.32)	0.870	1.09 (0.95–1.24)	0.217
	≥ 6 h	1.21 (1.03–1.42)	0.023	1.22 (0.89–1.68)	0.211	1.24 (1.08–1.43)	0.003
**Chronic diseases**	No	Ref		Ref		Ref	
	Yes	1.59 (1.38–1.82)	< 0.001	1.58 (1.22–2.05)	0.001	1.40 (1.25–1.58)	< 0.001

Insomnia showed the strongest association with CAD (OR = 14.64, 95% CI: 12.49–17.15, *p* < 0.001). In addition, two behavioral factors were independently associated with CAD in a graded manner: dietary regularity (more regular: OR = 1.55, 95% CI: 1.28–1.87; irregular: OR = 2.96, 95% CI: 2.45–3.58; both *p* < 0.001) and daily electronic device use (4–6 h: OR = 1.25, 95% CI: 1.04–1.49; > 6 h: OR = 1.78, 95% CI: 1.51–2.11). Modest but statistically significant increases in the odds of CAD were additionally observed for female sex (OR = 1.19, 95% CI: 1.04–1.37), university education or above (OR = 1.21, 95% CI: 1.01–1.45), and current drinking (OR = 1.23, 95% CI: 1.04–1.45).

Conversely, older age was associated with progressively lower odds of CAD (30–44 years: OR = 0.75, 95% CI: 0.63–0.90; 45–59 years: OR = 0.59, 95% CI: 0.48–0.73; ≥ 60 years: OR = 0.41, 95% CI: 0.31–0.55; all *p* < 0.001). Being married or cohabiting (OR = 0.77, 95% CI: 0.65–0.91), a monthly household income of at least 6000 RMB per capita (OR = 0.76, 95% CI: 0.64–0.90), occasional or regular exercise (OR = 0.71, 95% CI: 0.61–0.83 and OR = 0.72, 95% CI: 0.58–0.90, respectively), and 3000–6999 daily steps (OR = 0.76, 95% CI: 0.63–0.91) were also inversely associated with CAD (all *p* < 0.05). Sedentary time showed a nonlinear association: relative to less than 1 h/day, 1–5 h/day was associated with lower odds (OR = 0.80, 95% CI: 0.68–0.95), whereas 6 h or more per day was associated with higher odds (OR = 1.21, 95% CI: 1.03–1.42).

District, smoking status, and BMI were not retained in the final stepwise model. Estimates for anxiety and depression alone are presented in the corresponding columns of Table 3.

### 3.3 Alignment Diagram

Based on the multivariable logistic regression model, an alignment diagram was constructed to visualize the estimated probability of CAD (Figure 2). Each variable contributes a specific number of points according to its category. Insomnia showed the largest contribution, followed by irregular diet, female, physical chronic disease, and other risk factors. The total points are summed on the “Total Points” axis, and a vertical line drawn to the “Comorbidity Risk” axis provides the estimated probability of CAD for an individual.

**FIGURE 2:**
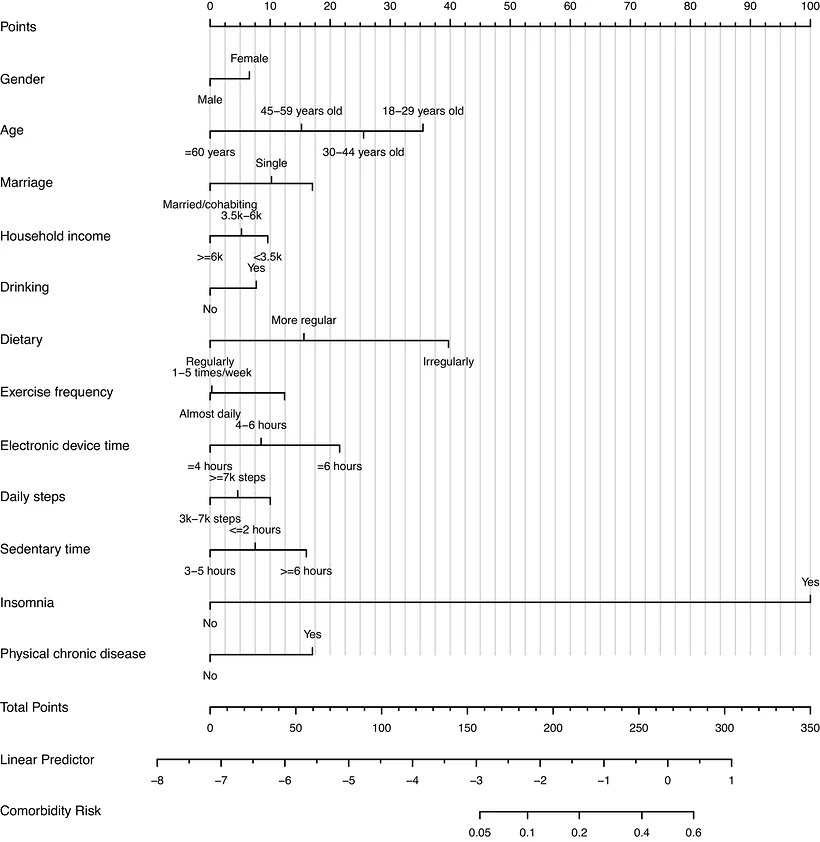
Alignment diagram for estimating the probability of comorbid anxiety and depression (CAD).

### 3.4 Decision Tree Model

The decision tree model was constructed using the CART algorithm, including all candidate variables. Variable importance (total Gini decrease, scaled to 100%) ranked insomnia first (100%), followed by dietary habits (22.07%), electronic device time (18.19%), sedentary time (17.02%), physical chronic disease (15.87%), and marriage (14.81%) (Table S3). These results indicate that insomnia and diet were the primary drivers of the tree structure, consistent with the logistic regression findings (Figure 3).

**FIGURE 3:**
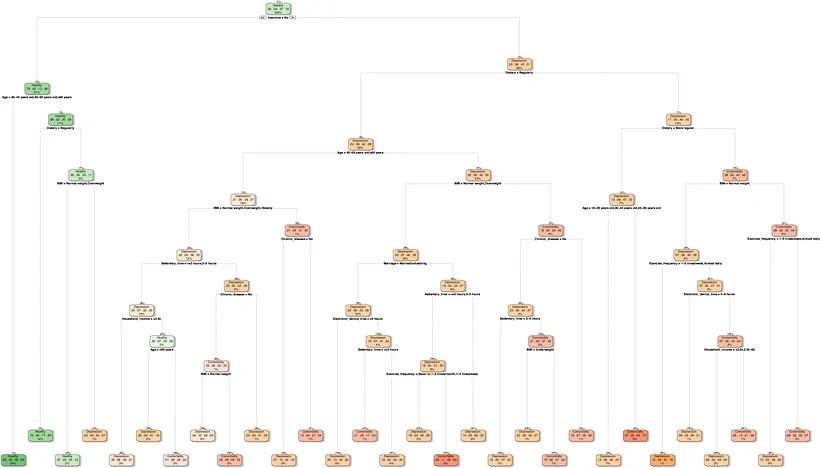
Exploratory decision tree for visualizing risk stratification of comorbid anxiety and depression (CAD).

### 3.5 Model Performance and Calibration

Figure 4 shows the ROC curves of the logistic regression model and the decision tree. The logistic regression model achieved an AUC of 0.866 (95% CI: 0.845–0.886), with a sensitivity of 0.830 and a specificity of 0.792. The decision tree model had an AUC of 0.839 (95% CI: 0.818–0.860), with a sensitivity of 0.865 and a specificity of 0.718. DeLong's test for two correlated ROC curves showed that the logistic regression model significantly outperformed the decision tree (AUC difference = 0.026, 95% CI: 0.012–0.041, *p* < 0.001). Calibration of the logistic regression model was assessed using the Hosmer–Lemeshow test (*χ* ^2^ = 2.09, df = 3, *p* = 0.553), indicating good agreement between observed outcomes and model‐estimated probabilities (Figure S1).

**FIGURE 4:**
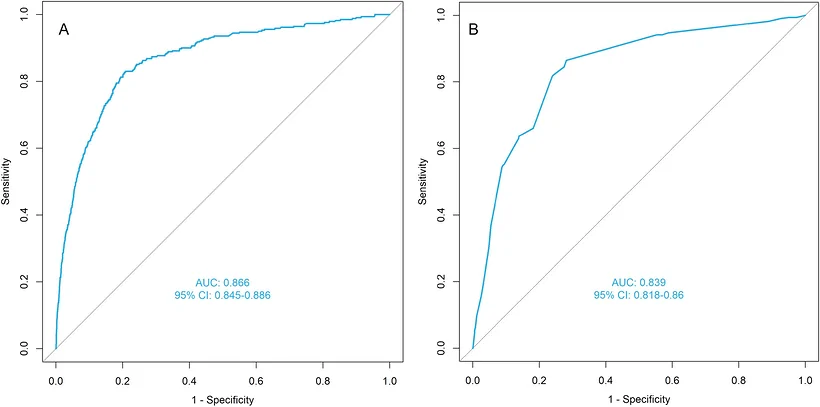
Receiver operating characteristic (ROC) curves of the logistic regression model and the exploratory decision tree model for comorbid anxiety and depression (CAD): (A) logistic regression model; (B) decision tree model.

### 3.6 Sensitivity Analysis

Sensitivity analyses supported the robustness of these findings (Tables S4–S6). After excluding all participants who reported any mental or sleep‐related condition (*n* = 48,264), physical chronic disease remained associated with CAD (adjusted OR = 1.43, 95% CI: 1.21–1.70), anxiety (OR = 1.62, 95% CI: 1.20–2.19), and depression (OR = 1.38, 95% CI: 1.20–1.58). The findings were also consistent across alternative definitions of the exposure: when all reported conditions in G15 question were considered, the OR for CAD was 2.16 (95% CI: 1.90–2.47), and in the four‐category analysis, both physical conditions only (OR = 1.37, 95% CI: 1.16–1.63) and somatic plus mental/sleep conditions (OR = 4.38, 95% CI: 3.57–5.37) were associated with higher odds than having neither condition. When the individual diseases were examined separately, 9 of the 15 conditions were associated with higher odds of CAD, most notably cataract or glaucoma (OR = 3.39, 95% CI: 1.77–6.49), chronic hepatitis or cirrhosis (OR = 2.53, 95% CI: 1.52–4.21), cardiovascular disease (OR = 2.43, 95% CI: 1.68–3.51), chronic gastritis or ulcer (OR = 2.23, 95% CI: 1.77–2.81), and intervertebral disk disease (OR = 2.07, 95% CI: 1.67–2.56), whereas hypertension, diabetes, and cerebrovascular disease were not significantly associated.

## 4 Discussion

In recent years, the comorbidity of depressive and anxiety disorders has become a research focus. In this large cross‐sectional study, we examined the prevalence and associated factors of CAD in adults. To our knowledge, this is one of the few studies using a large, population‐based sample to investigate CAD in the general adult population in China.

In our sample, the prevalence of CAD was 2.23%, which was lower than that reported in other studies involving specific patient populations. In an investigation conducted by Niu Yaqian et al. in Hefei in 2019, the detection rates of anxiety and depression comorbidity among nursing students at baseline and follow‐up were 16.9% and 18.2%, respectively (Niu et al. 2023). In 2023, the prevalence of anxiety and depression among college students in Anhui and Jiangxi Provinces was 16.9% (M. Wang, Tao, et al., 2023). In western China, the CAD prevalence in participants of 50 years and older was 9.0%. The occurrence of the above may be related to factors such as geographical characteristics, the age group composition of the sample, the type of questionnaire used, and the survey method. Furthermore, as Guangdong is a province with a robust economy, relatively high income levels, and a higher quality of life for its residents, this may contribute to a lower observed prevalence of CAD.

In this study, several factors were independently associated with CAD. The main findings are discussed below by category.

### 4.1 Impact of Basic Demographic Information Variables on CAD

In this study, the incidence of CAD decreased with increasing age. This result aligns with another cross‐sectional study conducted in western China (Liu et al. 2022). However, there were different opinions in other articles. A study that analyzed data from the Chinese Longitudinal Healthy Longevity Survey (CLHLS) reported that older age was associated with a lower likelihood of belonging to the 'severe depression and anxiety' class than to the 'low symptoms of depression and anxiety' class (Yu and Chen 2024). But other studies reported that there existed a linear increase in the prevalence of depression with increasing age (Zhao et al. 2024; W. Yu and Zhang 2023; Xiang et al. 2024). With age, older adults are at increased risk of chronic diseases and are more likely to suffer from depression and anxiety (Zhao et al. 2024). This may be attributed to the varying age stratification criteria employed in these studies (W. Yu and Zhang 2023), or it could be that as individuals age, their lives tend to stabilize, leading to improved mental health due to a more consistent quality of life. The present study suggests that the younger and middle‐aged groups of the adult population are more likely to suffer from CAD than the older age groups are, as opposed to suffering from depression and anxiety alone (W. Tan et al. 2020). It may be that the general economic situation in Guangdong is better, the old‐age care conditions of the elderly population are improved, whereas young people are under intense competition and pressure.

In univariable analysis, women had a higher crude risk of CAD, and this association remained significant after multivariable adjustment (OR = 1.19, 95% CI: 1.04–1.37), independent of lifestyle and socioeconomic factors. A similar pattern was observed when anxiety and depression were considered separately (OR = 1.58, 95% CI: 1.20–2.08 and OR = 1.17, 95% CI: 1.05–1.32, respectively). These findings are consistent with previous studies reporting a higher prevalence of psychological problems among women than among men (Liu et al. 2022; Peng et al. 2023), which may be attributed to women's greater sensitivity to the external environment and personal emotions, as well as the dual pressures of family responsibilities, childbearing, and career competition in modern society (Chiao and Chen 2018; Tuxunjiang et al. 2023).

Regarding marital status, being married or cohabiting was associated with lower odds of CAD compared with being single (OR = 0.77, 95% CI: 0.65–0.91). A pooled analysis of 106,556 adults from seven countries, including China, found that unmarried individuals had a substantially higher risk of depressive symptoms than married individuals (pooled OR = 1.86, 95% CI: 1.61–2.14) (Zhai et al. 2024); this protective effect is thought to operate through the exchange of social support between partners, greater economic security, and spouses' mutual influence on health behaviors (Zhai et al. 2024). Consistent with the notion that the absence of a partner carries risk, a meta‐analysis of 42 studies reported a high prevalence of both depression (19.2% by diagnostic criteria) and anxiety disorders (26.9%) among widowed individuals (Blanner Kristiansen et al. 2019). Although being divorced or widowed was not significantly associated with CAD in our sample (OR = 1.25, 95% CI: 0.93–1.68), this subgroup accounted for only 4.3% of participants, and the estimate was correspondingly imprecise.

### 4.2 Effect of Low‐Level Physical Activity Status on CAD

In this study, individuals who spent an average of 6 h or more per day using electronic devices, as well as those who remained sedentary for 6 h or more per day, exhibited a significantly greater risk of CAD compared to the non‐comorbidity group. Moreover, this effect intensified with prolonged exposure.

From work to play, the use of electronic devices such as cell phones and computers accounts for a large portion of modern people's lives. A study on a group of college students revealed that prolonged use of electronic devices is more likely to lead to anxiety (Qu et al. 2021), and prolonged sedentary behavior also leads to an increased risk of mental illness (Kandola et al. 2020; Singh et al. 2023). The prolonged use of electronic devices leads to less time spent interacting with reality, which reduces the time spent on actual exercise, interpersonal interactions, hobbies, and other mental health benefits. In addition, while sedentary time is not entirely used for electronic device use, it also puts residents in a state of low physical activity levels, especially those who sit for more than 6 h/day, and the risk of comorbidity is greater than that of residents who sit for less time. Low levels of physical activity are associated with less time spent exercising, whereas studies have shown that increasing the amount of time spent interacting with real‐world environments can help relieve depression and anxiety (Skoumalova et al. 2020). Prolonged periods of low levels of physical activity, such as the use of electronic devices and sedentary behavior, are prone not only to the development of a variety of chronic diseases but also to psychological disorders.

### 4.3 Association Between Irregular Diet and CAD

An irregular diet has an important influence on many diseases, especially digestive diseases (Chen et al. 2023; Jacka et al. 2017). In this study, eating habits are at the second‐level node in the decision tree model and are important variables for determining whether residents suffer from CAD. In the nomogram, irregular diet contributed substantially more points than regular diet, indicating a stronger association with CAD risk. Recently, a randomized controlled trial comparing a nutrition‐focused group intervention with a social support control for the treatment of depression found that the two approaches showed no statistically significant difference in outcomes, suggesting that dietary interventions may have comparable efficacy to social support (Kipfer et al. 2025). In addition, irregular eating, including overeating, may represent a form of emotional eating that functions to cope with stress, anxiety, depression and other negative affective states (Gabriel et al. 2023). Although research on the mechanism of CAD is not perfect (E. Zhang et al. 2024), it also suggests that good eating habits and a reasonable dietary composition have positive effects on mental illness. An analysis of a cohort study on the health of civil aviation in China revealed that eating time, the eating window period, and eating jet lag were related to changes in depression risk (Zhen et al. 2020), and this study revealed that eating irregularity was associated with higher odds of CAD through decision tree and alignment diagram models. A regular diet is not only essential for residents to develop good rest patterns but also a critical guarantee of physical health. This indicates that we can encourage residents to enhance their awareness of dietary regulations, focus on improving their quality of life, and prioritize mental health through diverse channels.

### 4.4 Physical Chronic Illnesses Associated With CAD

In this study, the presence of physical chronic diseases was associated with CAD, and this association remained consistent in the sensitivity analysis restricted to participants free of any mental or sleep‐related condition. Previous studies have confirmed the association between CAD and chronic diseases (Zhao et al. 2024; Liu et al. 2021; M. Tan et al. 2025; D. Wu et al. 2022; Z. Wang, Ma, et al. 2023). With the accelerated pace of modern life and increased stress in the population, chronic diseases are gradually showing an increasing trend (Liao et al. 2019). The influence of chronic diseases on CAD may be due to the complex etiology and long duration of the related chronic diseases, which are difficult to cure and cause disturbances to patients in their daily lives, making it difficult for them to relieve their depressed moods and thus induce CAD (Liao et al. 2019). This suggests that we should provide them with concise and convenient mental health identification guidance so that residents can raise awareness of their own mental health, timely detection, and early diagnosis and treatment.

### 4.5 Insomnia Is Closely Related to CAD

Insomnia was the strongest associated factor in both the logistic regression (OR = 14.64, 95% CI: 12.49–17.15) and the decision tree (first split variable). Although other modifiable factors, including irregular diet and prolonged electronic device use, also contributed to the risk stratification, their influence was considerably smaller, suggesting that sleep disturbance is the dominant discriminator of CAD risk in this population. This finding highlights the importance of sleep, together with lifestyle factors, in relation to mental health, and suggests that routine screening for insomnia symptoms could help identify individuals at high risk of CAD in community settings. However, due to the cross‐sectional design, these associations should not be interpreted as causal or predictive.

### 4.6 Research Limitations and Future Research Directions

This study is a cross‐sectional study that can illustrate only the correlation between the influencing factors and CAD but cannot establish a causal relationship. In addition, this study was completed in the form of a questionnaire, which may introduce some information bias, and further detailed research can be conducted on these influencing factors in the future to investigate whether they have an impact on CAD. Furthermore, chronic conditions were identified from a self‐reported, physician‐diagnosed checklist rather than from hospital records or ICD codes, and objective measurements such as blood pressure, lipid profile, renal function, and medication use were not available; misclassification of self‐reported chronic disease therefore cannot be excluded. The subjects of this study were residents from specific regions in Guangdong Province, which may introduce geographical limitations. Therefore, all findings should be interpreted as cross‐sectional associations, not as predictors or causal factors. To validate these findings and explore the associations between CAD and related risk factors across the country, longitudinal studies are needed to evaluate temporal relationships.

## 5 Conclusion

This large cross‐sectional study identified insomnia, irregular diet, prolonged electronic device use, prolonged sedentary time, physical chronic disease and female sex as factors associated with higher odds of CAD, whereas older age, higher household income, being married or cohabiting and taking physical exercise were associated with lower odds. The logistic regression model showed good internal discriminative ability, and the decision tree provided an exploratory visualization of risk stratification. These findings should be interpreted as associations, not causal determinants. Prospective studies with external validation are needed to confirm these results and to explore potential mechanisms.

## Author Contributions

**Zhuoqun Chen**, **Jiaying Huang**, and **Yuying Wang** conceived and drafted the manuscript. Jiaying Huang, Yuying Wang, and **Tongjie Chen** contributed to data analysis. **Jingxian Chen** and **Ke Zhu** contributed to visualization and paper revision. **Wenyan Tan** was responsible for data curation and thevalidation of the data analysis. **Xin‐lin Chen** was involved in conceptualization, methodology, funding acquisition, supervision, project administration, and review and editing of the manuscript. All authors read and approved the final manuscript.

## Funding

This study was supported by the National Key Research and Development Program of China (No. 2023YFC3503002), the Guangzhou Science and Technology Program (No. 2024B03J1293), the Guangdong Basic and Applied Basic Research Foundation (No. 2022A1515220162), the National Social Science Foundation of China (No.19ZDA360) and Guangzhou University of Chinese Medicine (University‐Hospital Joint Project of Guangzhou University of Chinese Medicine, No. GZYFS2024G01; Young Top‐notch Talent Cultivation Project, Category A) . The funding agencies had no role in the design and execution of the study; the collection, management, analysis, and interpretation of the data; or the preparation, review, or approval of the manuscript.

## Ethics Statement

The authors assert that all procedures contributing to this work comply with the ethical standards of the relevant national and institutional committees on human experimentation and with the Helsinki Declaration. The study protocol was approved by the Research Ethics Committee of the Guangdong Provincial People's Hospital, Guangdong Academy of Medical Sciences (Reference number: KY‐Z‐2022‐063‐02).

## Consent

Not applicable.

## Conflicts of Interest

The authors declare no conflicts of interest.

## Supporting information




**Supplementary Material**: brb371843‐sup‐0001‐FigureS1.pdf

**Supplementary Material**: brb371843‐sup‐0002‐SuppMat.docx

## Data Availability

Research data are not shared.
