# Supplementary material for: An Analysis of Factors Influencing Comorbid Anxiety and Depression Among Adults in China: A Large‐Scale Cross‐Sectional Study
Source: Brain Behav. 2026 Oct 21;16(10):e71843. doi: 10.1002/brb3.71843 (PMC13686344; doi:10.1002/brb3.71843)
Supplement: Supplementary file 1 [file BRB3-16-e71843-s001.pdf]

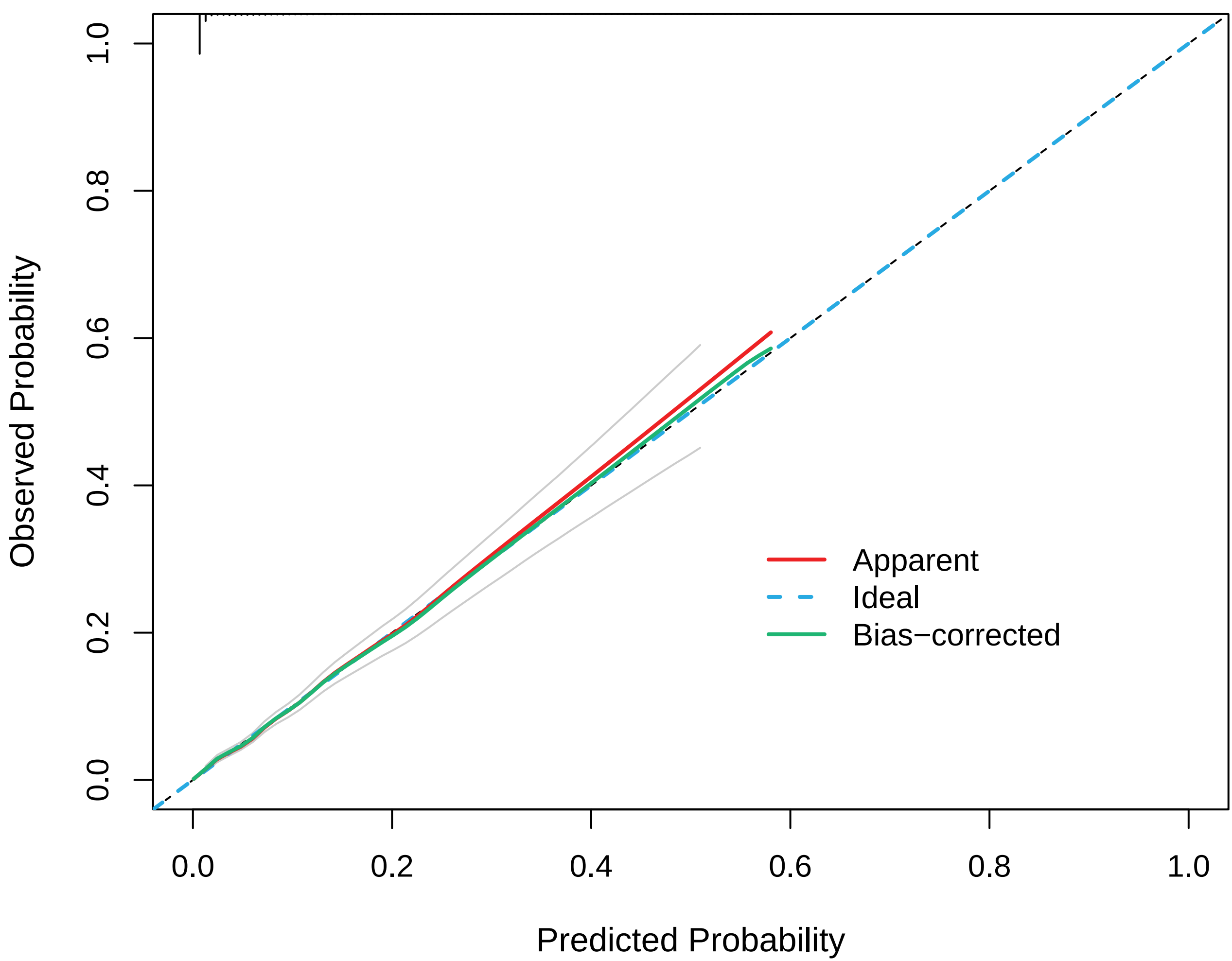

Observed Probability
Predicted Probability
0.0
0.2
0.4
0.6
0.8
1.0
Apparent
Ideal
Bias−corrected
