# Supplementary material for: An Analysis of Factors Influencing Comorbid Anxiety and Depression Among Adults in China: A Large‐Scale Cross‐Sectional Study
Source: Brain Behav. 2026 Oct 21;16(10):e71843. doi: 10.1002/brb3.71843 (PMC13686344; doi:10.1002/brb3.71843)
Supplement: Supplementary file 2 [file BRB3-16-e71843-s002.docx]

**Supplementary Material**

**Supplementary Box S1. Questionnaire item G15**

G15. In the past year, have you been clinically diagnosed with any of the following chronic diseases? (Multiple choices allowed)

0 = None of the following chronic diseases
1 = Hypertension
2 = Diabetes
3 = Cardiovascular disease (e.g., coronary heart disease)
4 = Cerebrovascular disease (e.g., stroke, cerebral infarction, cerebral thrombosis)
5 = Insomnia
6 = Obstructive sleep apnea syndrome
7 = Parkinson’s disease (if yes, diagnosed ______ years ago; whether received Parkinson’s disease-related treatment: ① Yes ② No)
8 = Dementia / Alzheimer’s disease
9 = Narcolepsy / hypersomnia
10 = Epilepsy
11 = Arthritis
12 = Intervertebral disc disease
13 = Chronic gastroenteritis / ulcer
14 = Gout
15 = Urinary system stones
16 = Chronic hepatitis / cirrhosis
17 = Cataract / glaucoma
18 = Gallstones / cholecystitis
19 = Depression
20 = Anxiety disorder
21 = Bipolar disorder
22 = Schizophrenia
23 = Sleep disorder
24 = Post-traumatic stress disorder
25 = Panic disorder
26 = Chronic obstructive pulmonary disease (COPD)
27 = Anemia
28 = Hyperlipidemia
29 = Other major disease (e.g., brain tumor), please specify: ______

**Table S1. Type III likelihood-ratio chi-square tests from the multinomial logistic regression model**

| **Variable** | **LR Chi‑square** | **df** | ***P* value** |
| --- | --- | --- | --- |
| Gender | 21.9 | 3 | <0.001 |
| Age | 108.7 | 9 | <0.001 |
| Education | 23.2 | 6 | <0.001 |
| Drinking | 10 | 3 | 0.018 |
| Dietary habits | 180.2 | 6 | <0.001 |
| Marriage | 50.6 | 6 | <0.001 |
| Household income | 38.4 | 6 | <0.001 |
| Exercise frequency | 58 | 6 | <0.001 |
| Electronic device time | 68.5 | 6 | <0.001 |
| Daily steps | 19.2 | 6 | 0.004 |
| Sedentary time | 33.4 | 6 | <0.001 |
| Insomnia | 3396.7 | 3 | <0.001 |
| Chronic disease | 73.7 | 3 | <0.001 |
| df :degrees of freedom |  |  |  |

**Table S2. Generalized variance inflation factors (GVIF) for independent variables in the multinomial logistic regression model**

| **Variable** | **GVIF** | **Df** | **GVIF^(1/(2*Df))** |
| --- | --- | --- | --- |
| Gender | 1.248551818 | 1 | 1.117386154 |
| Age | 2.115588545 | 3 | 1.133022500 |
| Education | 1.543422426 | 2 | 1.114605551 |
| Marriage | 1.556026642 | 2 | 1.116874196 |
| Household income | 1.177167507 | 2 | 1.041620615 |
| Drinking | 1.249340042 | 1 | 1.117738808 |
| Dietary | 1.225266134 | 2 | 1.052101422 |
| Exercise frequency | 1.205835790 | 2 | 1.047905313 |
| Electronic device time | 1.343645192 | 2 | 1.076641586 |
| Daily steps | 1.120115477 | 2 | 1.028763861 |
| Sedentary time | 1.393233705 | 2 | 1.086440616 |
| Insomnia | 1.051497475 | 1 | 1.025425509 |
| Chronic diseases | 1.157165765 | 1 | 1.075716396 |

**Table S3. Variable importance ranking in the decision tree model (CART)**

| **Variable** | **Importance** | **Relative_Importance** |
| --- | --- | --- |
| Insomnia | 8486.406392 | 100 |
| Dietary | 1873.061918 | 22.07132008 |
| Electronic device time | 1543.302451 | 18.18558268 |
| Sedentary time | 1444.679827 | 17.023458 |
| Chronic disease | 1346.652938 | 15.86835317 |
| Marriage | 1257.24683 | 14.81483177 |
| Age | 525.710802 | 6.19473989 |
| BMI | 266.925442 | 3.14532948 |
| Exercise frequency | 124.926884 | 1.47208227 |
| Household income | 60.047945 | 0.70757801 |
| Gender | 25.693 | 0.30275476 |
| District | 6.610816 | 0.07789888 |
| Drinking | 5.580973 | 0.06576367 |
| Daily steps | 5.1288 | 0.06043548 |

**Table S4. Sensitivity analysis of the association between physical chronic disease and mental health outcomes after excluding all participants reporting any mental or sleep-related condition (analysis sample, n = 48,264).**

| **Outcome** | **n** | **Events, n (%)** | **Crude OR (95% CI)** | ***P*** | **Adjusted OR (95% CI)** | ***P*** |
| --- | --- | --- | --- | --- | --- | --- |
| Comorbid anxiety and depression | 46,751 | 786 (1.7) | 1.44 (1.24–1.68) | <0.001 | 1.43 (1.21–1.70) | <0.001 |
| Anxiety | 46,184 | 219 (0.5) | 1.72 (1.30–2.27) | <0.001 | 1.62 (1.20–2.19) | 0.002 |
| Depression | 47,259 | 1,294 (2.7) | 1.37 (1.21–1.54) | <0.001 | 1.38 (1.20–1.58) | <0.001 |

Physical chronic disease was defined as reporting at least one of the 19 remaining physical conditions after excluding 10 mental and sleep-related conditions. All participants reporting any of these 10 conditions were removed for this analysis (n = 3,510: 1,469 with both physical and mental/sleep conditions and 2,041 with mental/sleep conditions only). All models were adjusted for sex, age, educational level, marital status, household income, drinking, dietary habits, exercise frequency, electronic device time, daily steps, sedentary time and insomnia. Adjusted estimates were derived from multinomial logistic regression with the healthy group as the reference category.

**Table S5. Association between chronic disease and mental health outcomes according to alternative definitions of the chronic disease exposure.**

| **Exposure definition** | **Participants, n (%)^a^** | **Comorbid anxiety and depression** | **Anxiety** | **Depression** |
| --- | --- | --- | --- | --- |
| **A. Binary definitions (reference: none of the conditions)** | | | | |
| All 29 conditions in item G15 (any vs none) | 15,065 (29.1) | 2.16 (1.90–2.47) | 2.05 (1.59–2.63) | 1.67 (1.50–1.87) |
| Physical chronic disease, 19 items (vs none)b | 13,024 (25.2) | 1.59 (1.38–1.82) | 1.58 (1.22–2.05) | 1.40 (1.25–1.58) |
| **B. Four-category definition (reference: neither condition)** | | | | |
| Neither condition (reference) | 36,709 (70.9) | 1.00 (reference) | | |
| Physical conditions only | 11,555 (22.3) | 1.42 (1.20–1.68) | 1.67 (1.24–2.24) | 1.36 (1.19–1.56) |
| Mental or sleep-related conditions onlyc | 2,041 (3.9) | 2.92 (2.42–3.53) | 2.77 (1.90–4.03) | 2.05 (1.72–2.43) |
| Both physical and mental/sleep conditions | 1,469 (2.8) | 4.38 (3.57–5.37) | 3.05 (1.97–4.73) | 2.62 (2.16–3.18) |

Values are adjusted odds ratios (95% CI). All P < 0.001. Outcomes were each compared with the healthy group (comorbid anxiety and depression, PHQ-9 ≥ 10 and GAD-7 ≥ 10; anxiety, GAD-7 ≥ 10 only; depression, PHQ-9 ≥ 10 only). All models were adjusted for sex, age, educational level, marital status, household income, drinking, dietary habits, exercise frequency, electronic device time, daily steps, sedentary time and insomnia.

a Counts and percentages are based on the full cohort (N = 51,774).

b Item G15 of the questionnaire lists 29 conditions; the 10 mental and sleep-related conditions (insomnia, obstructive sleep apnea syndrome, narcolepsy/hypersomnia, depression, anxiety disorder, bipolar disorder, schizophrenia, sleep disorder, post-traumatic stress disorder and panic disorder) were excluded from the primary definition of physical chronic disease.

c Estimates for this group are presented for completeness; because these conditions overlap conceptually with the outcomes, they should be interpreted with caution.

**Table S6. Prevalence of individual physical chronic diseases and their association with anxiety, depression and comorbid anxiety–depression.**

| **Disease** | **Healthy (n = 48,657)** | **Anxiety (n = 285)** | **Depression (n = 1,677)** | | **Comorbidity (n = 1,155)** | **Adjusted OR (95% CI) for anxiety** | **Adjusted OR (95% CI) for depression** | **Adjusted OR (95% CI) for comorbidity** |
| --- | --- | --- | --- | --- | --- | --- | --- | --- |
| Hypertension | 4749 (9.8) | 27 (9.5) | 136 (8.1) | | 89 (7.7) | 1.30 (0.84–2.01)  *P* = 0.240 | 1.22 (1.00–1.49)  *P* = 0.051 | 1.12 (0.87–1.43)  *P* = 0.375 |
| Diabetes | 1678 (3.4) | 6 (2.1) | 49 (2.9) | | 36 (3.1) | 0.71 (0.31–1.62)  *P* = 0.413 | 1.16 (0.85–1.58)  *P* = 0.342 | 1.21 (0.85–1.73)  *P* = 0.297 |
| Cardiovascular disease | 670 (1.4) | 9 (3.2) | 37 (2.2) | | 38 (3.3) | **2.31 (1.15–4.64)**  *P* **= 0.019** | **1.68 (1.17–2.41)**  *P* **= 0.005** | **2.54 (1.76–3.66)**  **<0.001** |
| Cerebrovascular disease | 255 (0.5) | 2 (0.7) | 22 (1.3) | | 10 (0.9) | 1.37 (0.33–5.66)  *P* = 0.663 | **2.68 (1.67–4.30)**  **<0.001** | 1.65 (0.85–3.20)  *P* = 0.143 |
| Arthritis | 1384 (2.8) | 14 (4.9) | 79 (4.7) | | 62 (5.4) | 1.49 (0.86–2.60)  *P* = 0.158 | **1.50 (1.17–1.92)**  ***P* = 0.002** | **1.67 (1.26–2.21)**  **<0.001** |
| Intervertebral disc disease | 1808 (3.7) | 19 (6.7) | 110 (6.6) | | 120 (10.4) | 1.26 (0.78–2.04)  *P* = 0.335 | **1.29 (1.04–1.59)**  *P* **= 0.021** | **2.07 (1.67–2.56)**  **<0.001** |
| Chronic gastritis/ulcer | 1143 (2.3) | 25 (8.8) | 100 (6.0) | | 101 (8.7) | **2.48 (1.62–3.79) <0.001** | **1.55 (1.24–1.94) <0.001** | **2.23 (1.77–2.81) <0.001** |
| Gout | 974 (2.0) | 9 (3.2) | 60 (3.6) | | 44 (3.8) | 1.37 (0.69–2.71)  *P* = 0.366 | **1.36 (1.02–1.81)**  *P* **= 0.037** | 1.38 (0.99–1.92)  *P* = 0.060 |
| Urinary calculus | 579 (1.2) | 8 (2.8) | 31 (1.8) | | 26 (2.3) | 1.79 (0.87–3.69)  *P* = 0.112 | 1.09 (0.74–1.60)  *P* = 0.674 | 1.26 (0.82–1.93)  *P* = 0.285 |
| Chronic hepatitis/cirrhosis | 167 (0.3) | 3 (1.1) | 21 (1.3) | | 20 (1.7) | 1.85 (0.58–5.90)  *P* = 0.301 | **1.97 (1.21–3.20)**  ***P* = 0.007** | **2.53 (1.52–4.21)**  **<0.001** |
| Cataract/glaucoma | 154 (0.3) | 1 (0.4) | 8 (0.5) | | 12 (1.0) | 1.05 (0.14–7.73)  *P* = 0.960 | 1.56 (0.74–3.30)  *P* = 0.240 | **3.39 (1.77–6.49)**  **<0.001** |
| Cholelithiasis/cholecystitis | 439 (0.9) | 4 (1.4) | 31 (1.8) | | 25 (2.2) | 1.02 (0.38–2.78)  *P* = 0.965 | 1.41 (0.96–2.09)  *P* = 0.082 | **1.63 (1.05–2.51)**  *P* **= 0.028** |
| Anaemia | 1206 (2.5) | 15 (5.3) | 108 (6.4) | | 82 (7.1) | 1.18 (0.69–2.01)  *P* = 0.547 | **1.49 (1.19–1.86)**  **<0.001** | **1.54 (1.19–1.98)**  **<0.001** |
| Hyperlipidaemia | 872 (1.8) | 10 (3.5) | 54 (3.2) | | 39 (3.4) | 1.54 (0.81–2.95)  *P* = 0.191 | **1.47 (1.09–1.98)**  ***P* = 0.012** | **1.48 (1.05–2.10)**  ***P* = 0.027** |
| Other major disease | 197 (0.4) | 6 (2.1) | 15 (0.9) | 6 (0.5) | | **3.26 (1.41–7.54)**  ***P* = 0.006** | 1.33 (0.76–2.32)  *P* = 0.325 | 0.72 (0.31–1.67)  *P* = 0.441 |

Each disease was modelled separately as a binary exposure (yes vs no) to avoid collinearity arising from multimorbidity. All models were adjusted for sex, age, educational level, marital status, household income, drinking, dietary habits, exercise frequency, electronic device time, daily steps, sedentary time and insomnia — the same covariate set used in the primary multivariable model. Estimates reaching statistical significance (*P* < 0.05) are shown in bold.

Diseases with fewer than 150 cases in the cohort (Parkinson disease, dementia, epilepsy, chronic obstructive pulmonary disease) were not analysed because of insufficient numbers. Estimates for anxiety should be interpreted with caution given the small number of events.

**Figure S1.** **Calibration plot for the logistic regression model (Comorbidity vs. Healthy，Hosmer‑Lemeshow test *P* = 0.553)**
